# The extracellular matrix glycoprotein fibrillin-1 in health and disease

**DOI:** 10.3389/fcell.2023.1302285

**Published:** 2024-01-10

**Authors:** Li Li, Junxin Huang, Youhua Liu

**Affiliations:** ^1^ State Key Laboratory of Organ Failure Research, Division of Nephrology, Nanfang Hospital, Southern Medical University, Guangzhou, China; ^2^ National Clinical Research Center of Kidney Disease, Guangdong Provincial Institute of Nephrology, Guangzhou, China

**Keywords:** FBN1, extracellular matrix, marfan syndrome, TGF-β, chronic kidney diseases

## Abstract

Fibrillin-1 (FBN1) is a large, cysteine-rich, calcium binding extracellular matrix glycoprotein encoded by *FBN1* gene. It serves as a structural component of microfibrils and provides force-bearing mechanical support in elastic and nonelastic connective tissue. As such, mutations in the *FBN1* gene can cause a wide variety of genetic diseases such as Marfan syndrome, an autosomal dominant disorder characterized by ocular, skeletal and cardiovascular abnormalities. FBN1 also interacts with numerous microfibril-associated proteins, growth factors and cell membrane receptors, thereby mediating a wide range of biological processes such as cell survival, proliferation, migration and differentiation. Dysregulation of FBN1 is involved in the pathogenesis of many human diseases, such as cancers, cardiovascular disorders and kidney diseases. Paradoxically, both depletion and overexpression of FBN1 upregulate the bioavailability and signal transduction of TGF-β via distinct mechanisms in different settings. In this review, we summarize the structure and expression of FBN1 and present our current understanding of the functional role of FBN1 in various human diseases. This knowledge will allow to develop better strategies for therapeutic intervention of FBN1 related diseases.

## 1 Introduction

Fibrillin-1 (FBN1) is a large, cysteine-rich glycoprotein that plays a crucial role in the formation and function of elastic fibers in connective tissues. FBN1 is expressed during embryonic development and in adult tissues, and secreted and incorporated into the extracellular matrix (ECM) network that surrounds tissue resident cells (Carta et al., 2006). Structurally, FBN1 contains multiple epidermal growth factor (EGF)-like motifs arranged in tandem, transforming growth factor-β (TGF-β) binding modules (TB modules), which shows sequence homology to the TB modules of the latent TGF-β binding proteins (LTBPs), and a proline-rich region (Zeyer and Reinhardt, 2015; Du et al., 2021). FBN1 is a component of calcium-binding microfibrils and is involved in microfibril assembly, formation and assembly of ECM, maintenance of tissue homeostasis and provision of mechanical support to elastic and inelastic connective tissues (Schrenk et al., 2018). As such, FBN1 contributes to artery wall elasticity and regulates elastic fiber homeostasis. Genetic knockout mice with FBN1 deficiency (*Fbn1* ^*−/−*^) die shortly after birth due to aortic aneurysm formation and rupture (Carta et al., 2006), suggesting that FBN1 deposition is a prerequisite for blood vessel maturation and function during neonatal life. Mutations in the *FBN1* gene can cause a wide range of phenotypes that differ in their severity, including fetal death, developmental problems or Marfan syndrome, a connective tissue disorder characterized by pleiotropic manifestations involving primarily the ocular, skeletal and cardiovascular systems.

As an ECM protein, FBN1 also plays a fundamental role in regulating cell-cell and cell-matrix interactions in physiological and pathological settings. FBN1 binds other ECM proteins, growth factors and cell surface receptors, thereby mediating diverse biological processes such as organ development, tissue homeostasis and injury repair. Studies show that FBN1 controls the bioavailability of extracellular TGF-β, modulates cell behaviors, and regulates cell survival and differentiation (Jensen et al., 2012). Furthermore, FBN1 also directly interacts with various bone morphogenetic proteins (BMPs) (Gregory et al., 2005; Sengle et al., 2008; Weinbaum et al., 2008; Sengle et al., 2011; Wohl et al., 2016) and activates other signal pathways, such as the mitogen-activated protein kinase (MAPK) and signal transducer and activator of transcription (STAT) signaling (Wang Z. et al., 2022). FBN1 is often found to be dysregulated in several human diseases such as cancer, cardiovascular and kidney diseases. Under pathological conditions, dysregulated FBN1 can promote the proliferation of tumor cells or induce apoptosis in endothelial cells (Li et al., 2021; Wang X. et al., 2022). Increasing evidence suggests that the dysregulation of FBN1 also plays a pivotal role in the pathogenesis of a wide range of human disorders.

In this article, we aim to review the structure, expression and function of FBN1, and summarize the role and potential mechanism of FBN1 in the pathogenesis of many human diseases. A better understanding of the role and mechanism of action of FBN1 in different settings will help to delineate the logic behind human diseases and develop rational strategies for treatment.

## 2 Domain structure of FBN1

FBN1 is a large protein with 350 kDa in size. It serves as the primary structural constituent of microfibrils of 10–12 nm in diameter, which are ubiquitously present in all connective tissues (Zigrino and Sengle, 2019). The human *FBN1* gene resides on chromosome 15 long arm (15q15-21.1), spans about 230 kb genomic DNA, and is highly split into 65 exons, and codes for a protein with 2871 amino acids (Davis and Summers, 2012; Wang et al., 2018).

The structure of FBN1 consists of 47 six-cysteine EGF-like and 7 eight-cysteine TGF-β binding protein-like (TB) domains (Schrenk et al., 2018). Among the 47 EGF domains, it is noteworthy that 43 of these domains exhibit the occurrence of a calcium binding consensus sequence, hence being referred to as calcium binding EGF-like domains (cbEGF) (Zeyer and Reinhardt, 2015; Muthu and Reinhardt, 2020). The cbEGF domain performs a supportive role through limiting the mobility of interdomain areas and protecting molecules from proteolysis (Jensen et al., 2012). These cbEGF domains also interact with FBN2, as well as with other ECM proteins, such as fibulin-2, -4 and -5, heparin, microfibril-associated glycoprotein (MAGP)-1, aggrecan and versican (Reinhardt et al., 1996; Jensen et al., 2001; Isogai et al., 2002; Lin et al., 2002; Isogai et al., 2003; Thomson et al., 2019).

**FIGURE 1:**
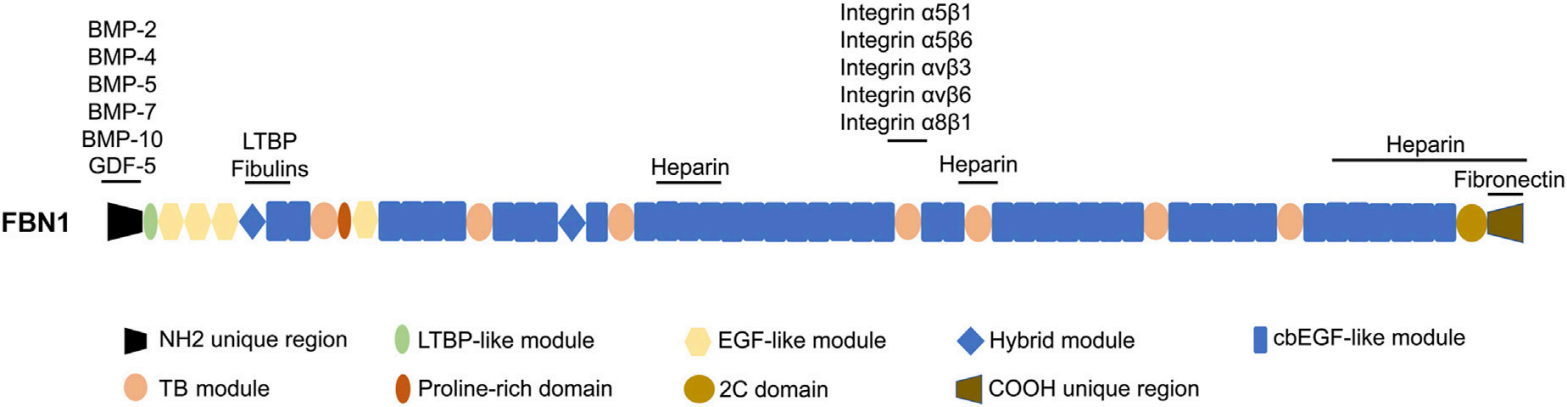
The domain structure of FBN1 and the sites responsible for interacting with other proteins. LTBP, latent TGF-β-binding protein; cbEGF, calcium binding EGF; EGF-like, epidermal growth factor-like; 2C, 2-cysteine; BMP-2, bone morphogenetic protein 2; GDF-5, growth differentiation factor 5.

The disulfide bond that arises from the interaction between the six cysteine residues present within EGF and cbEGF (Smallridge et al., 2003) is helpful to stabilize FBN1. The cbEGF domain is observed to occur in numerous copies, and in the majority of instances, each cluster of cbEGF repeats is distinctly differentiated from the subsequent cluster by means of the TB domain. Furthermore, apart from the aforementioned module types, there exist 2 hybrid (hyb) domains that exhibit both sequence and structural resemblances to the cbEGF and TB domains. Additionally, it is worth noting that there exists a proline-rich region (Peeters et al., 2022). Figure 1 presents the domain structure of FBN1.

The region across the TB5 and cbEGF18 domains in FBN1 is commonly called the “neonatal” region due to its susceptibility to mutations that result in an extremely severe form of neonatal Marfan syndrome (Kainulainen et al., 1994). The TB4 domain includes an RGD (arginine-glycine-aspartic acid) motif that exhibits binding affinity towards integrins α5β1, α5β6, αvβ3, αvβ6, and α8β1 (Lee et al., 2004; Bax et al., 2007; Del Cid et al., 2019). Mutations in the region near the RGD binding site in FBN1 result in a state called stiff skin syndrome (SSS), and this domain is associated with a pathological state which is characterized by the excessive deposition of microfibers and fibrosis in the skin (Loeys et al., 2010b). Mice with an RGE knock-in within TB4 of FBN1 develop a phenotype of systemic scleroderma (Gerber et al., 2013). The vicinity surrounding the hyb1 domain of FBN1, particularly the interface between the EGF3 and hyb1 domains, serves as a binding site for fibulins and LTBPs (El-Hallous et al., 2007; Ono et al., 2009; Robertson et al., 2017). It is worth noting that FBN1 itself has seven variable affinity heparin binding sites (Cain et al., 2005; Cain et al., 2008; Yadin et al., 2013; Sabatier et al., 2014), which have the ability to interact with heparan sulfate chains. In addition, previous studies have demonstrated that certain growth factors, such as BMP-2, BMP-4, BMP-5, BMP-7, BMP-10, and growth differentiation factor 5 (GDF-5), exhibit varying affinities when binding to recombinant segments of FBN1 in the N-terminal region (Sengle et al., 2008; Sengle et al., 2011; Sengle et al., 2012; Spanou et al., 2023).

## 3 Expression pattern of FBN1

**FIGURE 2:**
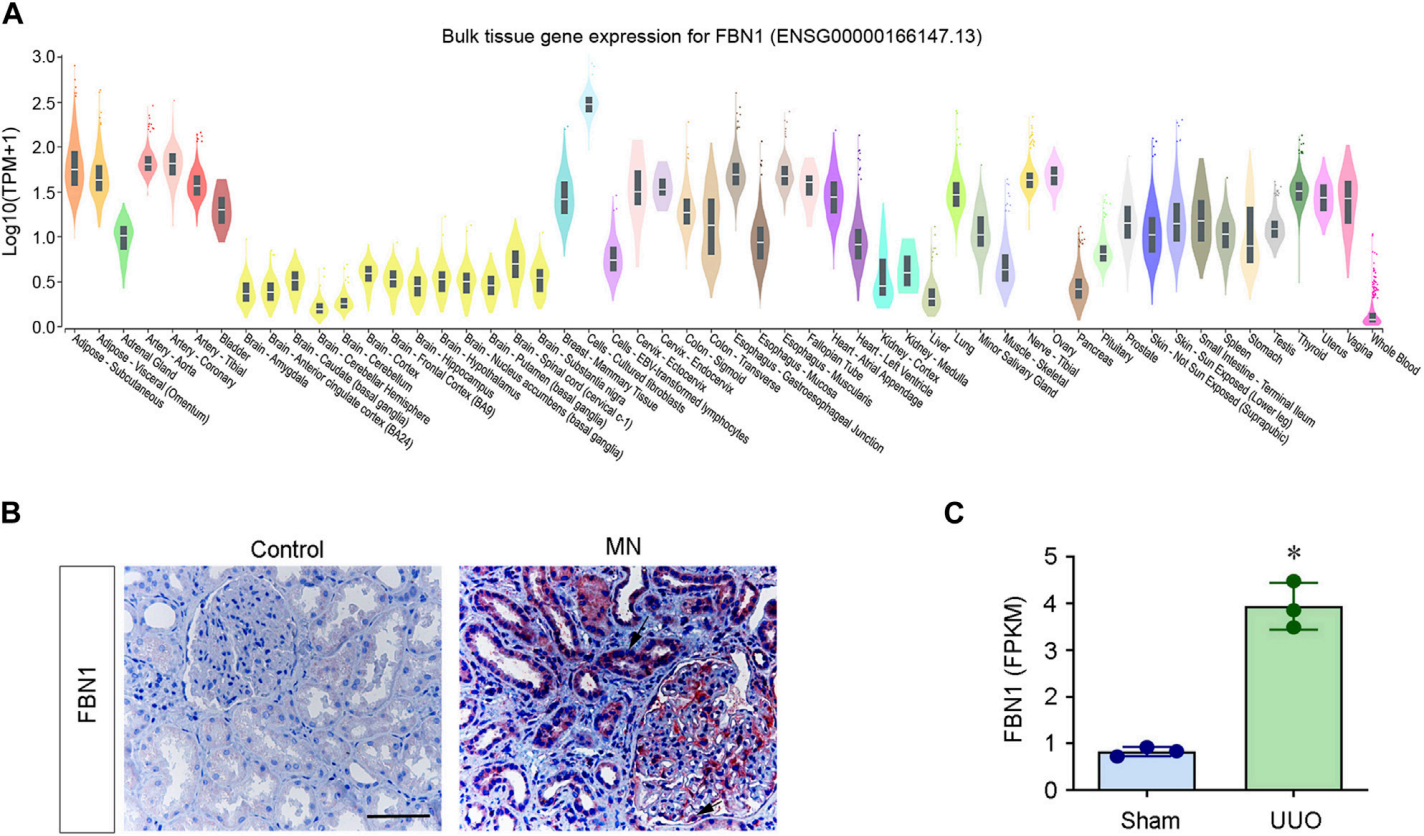
FBN1 mRNA expression in human tissues. **(A)** Human tissue and cell gene expression data were extracted from the online database https://www.gtexportal.org (dataset ENSG00000166147.13). The *Y*-axis represents the normalized expression values. **(B)** Immunohistochemical staining for FBN1. Renal expression and localization of FBN1 protein in various groups are depicted on representative micrographs. Control, nontumor kidney part obtained from individuals diagnosed with renal cell carcinoma; MN, membranous nephritis. Scale bar: 50 μm. Arrow indicates positive staining. **(C)** RNA-seq analysis shows the expression level (FPKM) of mouse FBN1 in normal (sham) and obstructed kidneys after unilateral ureteral obstruction (UUO). ^*^ *p* < 0.05. FPKM, fragments per kilobase per million mapped reads. Date are available at NCBI with accession number PRJNA846588.

FBN1 is expressed in both embryos and adult tissues. Studies using mouse models have confirmed the vital importance of FBN1 for vascular development and function. FBN1 null mice die perinatally because of ruptured aortic aneurysm (Carta et al., 2006). Consistent with its role in connective tissue, FBN1 is mainly expressed in interstitial cell types and tissues. Figure 2A shows the expression pattern derived from publicly available human microarray data (https://www.gtexportal.org). The expression of FBN1 is observed in a wide variety of tissues. As shown in Figure 2A, substantial mRNA expression of FBN1 is detected in cultured fibroblasts, subcutaneous adipose tissue, aorta artery, coronary artery, esophagus at the gastroesophageal junction, muscular esophagus, tibial nerve, and ovary. Notably, cultured fibroblasts display the highest level of expression among these tissues. However, the levels of FBN1 expression are relatively lower in many other tissues, including whole blood, cerebellum, cerebellar hemispheres, amygdala, anterior cingulate cortex, liver and pancreas.

The expression of FBN1 is clearly dysregulated in various pathological conditions. Particularly, FBN1 is often upregulated in a variety of cancers and has been proposed as a promising tumor biomarker. As summarized in Table 1, FBN1 is highly expressed in gastric cancer, colorectal cancer, osteosarcoma, papillary thyroid carcinoma, renal cell carcinoma and ovarian cancer.

**TABLE 1:** The expression and function of FBN1 across multiple human tumors.

Tumor	Expression	Function and mechanism	References
Gastric cancer	Upregulation	Activates TGF-β1 and PI3K/Akt signaling; promotes cell proliferation and tumor progression	Yang et al. (2017), Wang et al. (2022a)
Colorectal cancer	Increased methylation	FBN1 methylation is a clinically important biomarker for early diagnosis and screening	Guo et al. (2013), Lv et al. (2022)
Osteosarcoma	Upregulation	Increases osteosarcoma EMT, invasion and migration, and tumorigenesis	Liu et al. (2020)
Papillary thyroid carcinoma	Upregulation	Downregulating of FBN1 inhibits cell viability and colony formation *in vitro* and decreases tumor growth *in vivo*	Ma et al. (2016)
Metaplastic carcinoma	Upregulation	Enriched in metaplastic carcinoma of the breast with spindle sarcomatous metaplasia	Lien et al. (2019)
Renal clear cell carcinoma	Upregulation	High expression was associated with poor survival outcome	Chen et al. (2017)
Germ cell neoplasia	Upregulation	Be a factor in the genesis of germ cell tumors in situ	Cierna et al. (2016)
Ovarian cancer	Upregulation	Regulates glycolysis and angiogenesis through the VEGFR2/STAT2; promotes ovarian cancer metastasis	Wang et al. (2015), Wang et al. (2022b)
Endometrial cancer	Downregulation	Interacts with FBXO2, leading to polyubiquitinated degradation of FBN1	Che et al. (2020)

FBN1 is also induced in different organs after injury. In a periodontal disease model, FBN1 expression was significantly elevated at the onset of periodontal tissue destruction (Handa et al., 2018). In addition, FBN1 was significantly induced in biliary fibrosis and hepatic fibrosis (Dubuisson et al., 2001; Lamireau et al., 2002; Lorena et al., 2004). Recently, we conducted a proteomic profiling of ECM proteins in the context of fibrotic kidney. Our findings revealed that FBN1 was significant upregulated in the decellularized kidney tissue scaffold in chronic kidney disease (CKD). This observation suggests that FBN1 is a major constituent of the fibrotic microenvironment in the diseased kidney (Li et al., 2021; Li et al., 2022; Li et al., 2023). FBN1 is upregulated in many experimental models of CKD as well as in human biopsies obtained from individuals diagnosed with CKD (Figures 2B, C). In contrast to its origin from interstitial cells in physiological conditions, the production and secretion of FBN1 in CKD predominantly occur in the injured kidney tubular epithelial cells. These observations suggest an altered expression pattern of FBN1 in pathological setting.

## 4 FBN1 microfibril assembly

FBN1-containing microfibrils form the periphery of the elastic fiber, acting as a scaffold for the deposition of elastin. A complex process is involved in the assembly of FBN1 monomers into microfibrils. FBN1 initially forms microfilaments by interacting with itself and other proteins such as fibronectin and heparan acetyl sulfate proteoglycan (Baldwin et al., 2013; Godwin et al., 2019; Adamo et al., 2021). FBN1 monomers are assembled into microfibrils at the cell surface, with initial steps include preprotein processing, multimerization driven by the C-terminus, and alignment of neighboring molecules from head-to-tail (Jensen et al., 2014). A prerequisite for microfibril assembly is the processing of FBN1 by furin. Before or after the secretion of the precursor FBN1, furin mediates proteolytical cleavage at the N-terminal and C-terminal ends of FBN1, which would promote the terminal interaction and lead to axial and lateral self-assembly (Marson et al., 2005; Jensen et al., 2014; Schmelzer and Duca, 2022). The C-terminal of FBN1 condenses to form a bead-like structure by directly forming intermolecular disulfide bonds, which increases the apparent affinity of the C-terminal for the N-terminal fragments, and promotes the interaction with the N-terminal, as well as with fibronectin and heparan acetyl sulfate (Hubmacher et al., 2014; Jensen et al., 2014; Sabatier et al., 2014). The latest cryo-electron microscopy results show that the volume and mass data measured for the major bead formed by the N-terminal and C-terminal structural domains support the idea that the N-terminal and C-terminal have extensive overlap or interaction within this region (Godwin et al., 2023). As a result of the N-terminal and C-terminal interactions between FBN1 molecules, microfibrils are assembled from monomers by head-to-tail alignment of FBN1 monomers (Hubmacher et al., 2014). The N-terminal/C-terminal interaction is an important step in the microfibril assembly pathway, which mediates the end-to-end assembly of FBN1 monomers.

Heparan sulfate proteoglycan additionally regulates microfibril assembly. Because of the strong interaction with FBN1, heparan sulfate proteoglycan performs a key function in positioning FBN1 on the cell surface, enhancing homotypic N-terminal interactions, and promoting the stronger binding of FBN1 multimers and heparan sulfate proteoglycan (Cain et al., 2008; Jensen et al., 2014; Godwin et al., 2019). Microfibrils can also be further stabilized by cross-linking between N and C terminals mediated by glutamine aminotransferase. In addition, fibrillar assembly requires fibronectin (Godwin et al., 2019; Schmelzer and Duca, 2022). Multimerized C-terminal fragments of FBN1 have a high affinity for fibronectin, and fibronectin is essential for microfibril assembly (Jensen et al., 2014). Earlier investigations have showed the ability of fibronectin to interact with the C-terminal domain of FBN1 and promote microfibril assembly (Tsutsui et al., 2010). Once the microfibril is completely stabilized by glutamine transferase and disulfide bonds, microfibril assembly is independent of fibronectin (Sabatier et al., 2013).

The mechanism of microfibril assembly, however, may vary with cell type. Microfibril assembly in mesenchymal cells, including dermal fibroblasts or vascular smooth muscle cells, is contingent upon the existence of fibronectin networks. The process of microfibril assembly in retinal pigment epithelial cells occurs autonomously, without any reliance on fibronectin (Hubmacher et al., 2014; Hubmacher and Apte, 2015). It has also been suggested that the formation of microfibril is improbable to be just a self-assembly mechanism, and there exists certain substantiation indicating the contribution of cells in this process. Microfibril deposition in fibroblasts requires fibronectin RGD-dependent α5β1 integrins (Kinsey et al., 2008). Other proteins also interact with microfibril, including LTBPs (Isogai et al., 2003), MAGPs (Mecham and Gibson, 2015), a disintegrin and metalloproteinase with thrombospondin motifs (ADAMTS), ADAMTS-like (ADAMTSL) proteins and elastin (Brocker et al., 2009). In the formation of microfibril, FBN1 is involved in both the structural and regulatory aspects (Schmelzer and Duca, 2022), such as forming elastic fibril scaffolds, preserving the stability of elastic fibril, and making key contributions to tissue mechanics (Kielty and Shuttleworth, 1995; Sabatier et al., 2013; Thomson et al., 2019).

## 5 FBN1 and TGF-β modulation

One of the major functions of FBN1 is its ability to regulate TGF-β bioavailability and activation, which is also the mechanism behind FBN1 actions in various physiological and pathological conditions. The TGF-β isoforms, including TGF-β1, TGF-β2, and TGF-β3, are produced as precursor proteins. These precursor proteins consist of a growth factor domain located at the C-terminal and a latency associated peptide (LAP) situated at the N-terminal (Schrenk et al., 2018). The TGF-β molecule undergoes homodimerization and subsequently binds with LAP within the cytoplasm, resulting in the formation of small latency complexes (Davis and Summers, 2012). The interaction between the SLC and latent TGF-β-binding proteins (LTBP)-1, −3, and −4 occurs via disulfide bonding, resulting in the formation of a complex known as the large latency complex (LLC) (Davis and Summers, 2012). The linkage between the LLC and ECM is established by the reciprocal interaction between the C-terminal domain of LTBP-1 and the N-terminal domain of FBN1 (Isogai et al., 2003). The co-localization of LTBP1 and FBN1 has been demonstrated throughout the process of microfibril assembly (Massam-Wu et al., 2010). The LTBP4 exhibits a strong affinity for the N-terminal area of FBN1, although LTBP3 does not demonstrate binding to this specific region. This observation suggests the presence of additional binding sites within the FBN1 (Isogai et al., 2003). LTBP2 seems to be in competition with other LTBPs for binding to FBN1, although it does not seem to interact with LAP (Isogai et al., 2003; Hirani et al., 2007). Although FBN1 does not bind TGF-β or SLC directly, but these interactions with LTBP indicate that FBN1 contributes to the regulation of TGF-β (Massam-Wu et al., 2010; Lockhart-Cairns et al., 2022). For example, ECM disturbances that result in the extension or deformation of FBN1 molecule can be transferred to the prodomain of TGF-β via LTBP and allow LLC to release its active form (Shi et al., 2011; Sengle et al., 2012).

**FIGURE 3:**
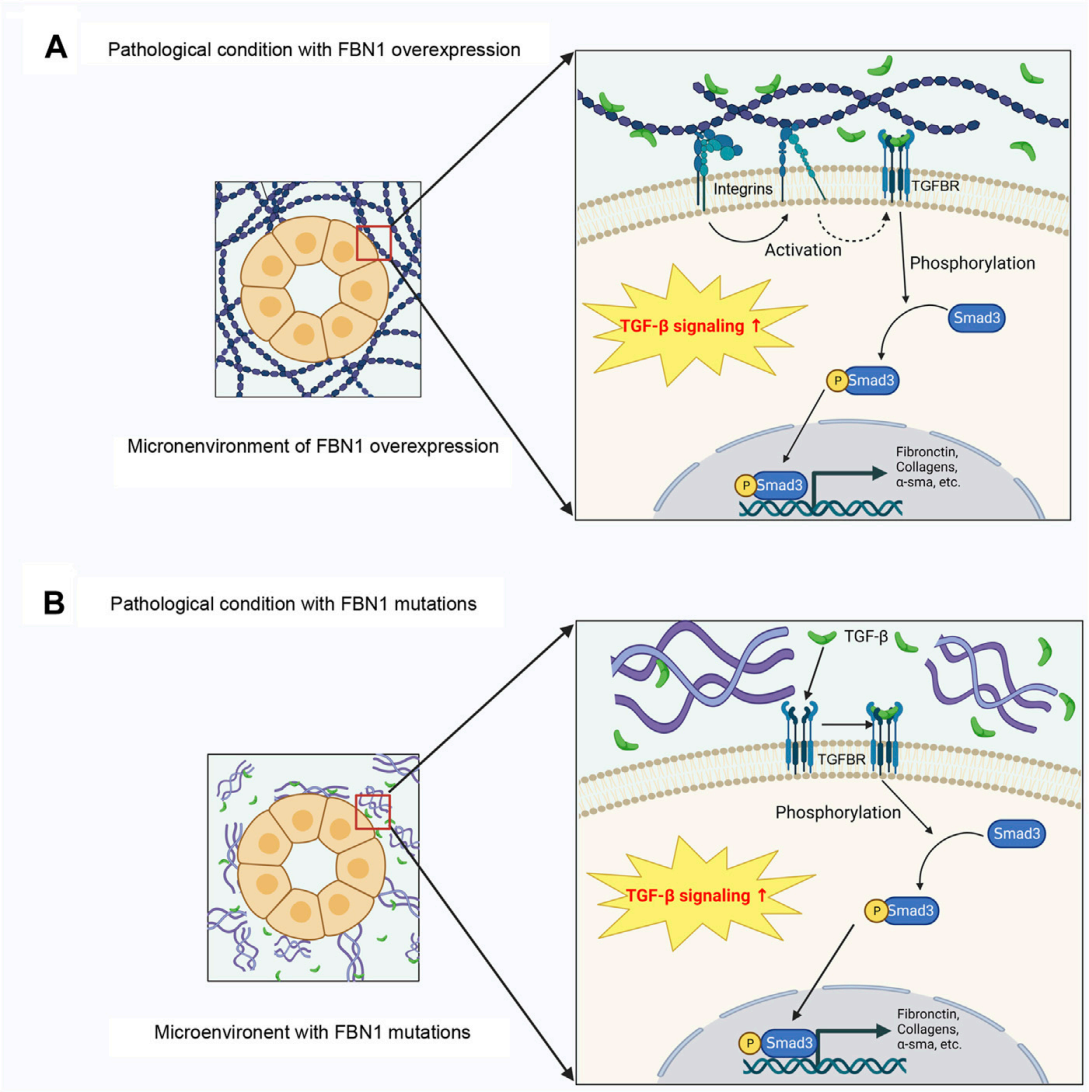
FBN1 paradoxically promotes TGF-β activation in different settings. **(A)** In various pathological conditions, FBN1 is induced, which leads to TGF-β1 activation through integrin signaling. Activate TGF-β then activates Smad signaling through membranous TGFBRs. **(B)** In the setting of genetic diseases, alterations in FBN1 gene result in the loss-of-function of FBN1, which liberates TGF-β release. Activated TGF-β promotes Smad-mediated gene expression. TGFBR, TGF-β receptor.

The stimulation of TGF-β signaling by the secretion of TGF-β from the LLC is facilitated by various ways, such as proteolysis by plasmin or matrix metalloproteinases (MMPs), or integrin binding-mediated LLC conformational changes and subsequent force dependent activation (Shi et al., 2011; Hinz, 2013; Hu et al., 2021; Peeters et al., 2022; Sun and Liu, 2022). The stimulation of TGF-β occurs via its binding to the TGF-β receptor 2 (TGFBR2), which subsequently triggers the phosphorylation of TGFBR1 (Yuan et al., 2022). The subsequent downstream events of signal transduction encompass the canonical or noncanonical pathway. Within the context of the canonical pathway, the process involves the phosphorylation of receptor-regulated Smad2/3, followed by the formation of a complex with Smad4 (Shi et al., 2011; Hinz, 2013; Peeters et al., 2022). Subsequently, the complex is translocated to the nucleus where it initiates the process of transcription for TGF-β target genes, such as collagen type 1 α1 chain, collagen type 3 α1 chain, and plasminogen activator inhibitor-1 (PAI-1), as well as 60 ECM-associated genes (Peng et al., 2022). The noncanonical TGF-β signaling activates ERK, p38 MAPK, and c-Jun N-terminal kinase (JNK), and finally stimulates the transcription of TGF-β target genes (Fu et al., 2017; Yuan et al., 2022). We recently show that in endothelial cells, FBN1 interacts with integrin αvβ6, leading to the stimulation of TGF-β precursor present in the extracellular space, without influencing total TGF-β abundance (Li et al., 2021). Therefore, upregulated FBN1 accumulates in the extracellular space and forms a special microenvironment, thereby resulting in TGF-β stimulation via αvβ6 integrins (Figure 3A).

FBN1 also directly interacts with a variety of BMPs, such as BMP-2, -4, -7 and -10 (Sengle et al., 2008; Sengle et al., 2011; Wohl et al., 2016), and osteoclastogenic cytokine receptor activator of nuclear factor κB ligand (RANKL) across multiple cellular microenvironments (Tiedemann et al., 2013). FBN1 can also indirectly regulate TGF-β signaling through its binding partner MAGP1. MAGP-1 could suppress LTBP-1 binding to FBN1, a process that is responsible for anchoring the active form of TGF-β to the microfibril structure (Weinbaum et al., 2008). Therefore, the reduction of MAGP1 will further lead to the stimulation of the TGF-β signaling.

Furthermore, an addition to FBN1 upregulation, loss or reduction of FBN1, paradoxically, also activates TGF-β signaling (Figure 3B). Reduction of FBN1 due to gene mutations induces TGF-β activation, resulting in the progression of fibrillinopathies (Granata et al., 2017). In order to substantiate this hypothesis, the injection of TGF-β antagonists has been observed to induce an anti-apoptotic effect in the pulmonary tissue of mice with FBN1 deficiency (Neptune et al., 2003). Moreover, the neutralization of TGF-β antibodies normalize TGF-β levels in MFS mouse models, and successfully prevent the development of aortic aneurysm (Ng et al., 2004). Moreover, TGF-β antagonists can reduce circulating TGF-β levels in individuals with MFS (Matt et al., 2009). It is worth noting that alterations in LTBPs or TGFBRs, as shown in Loyes Dietz syndrome, can potentially result in the unregulated secretion of TGF-β. Disturbance of TGF-β signal transduction is further found in other fibrillar diseases, like Stiff skin syndrome (Loeys et al., 2010b) and acromicor geleophysic dysplasia (Le Goff et al., 2011).

## 6 *FBN1* mutation and marfan syndrome

The *FBN1* gene exhibits high levels of expression within the vascular wall of large arteries, playing a significant role in processes such as vascular remodeling and the development of hereditary disorders (Schrenk et al., 2018). In this context, mutations in the *FBN1* gene can cause diseases of multiple organs, which are collectively called fibrillinopathies.

It is well known that mutations in the *FBN1* gene can cause MFS, which is an autosomal dominant disease affecting connective tissue. This condition is marked with various abnormalities in bone, ocular tissues and cardiovascular system (Ramirez et al., 2018). Skeletal characteristics encompass an elevated stature, disproportionate long limbs and spider like toes and fingers, chicken breasts and pectus excavatum caused by excessive growth of long bones, and other characteristics attributed to atypical bone development, such as thoracic lordosis, scoliosis, stenosis. Lens dislocation and subluxation due to ciliary lobule defects are the hallmark characteristics of MFS (Ramachandra et al., 2015; Du et al., 2021). Furthermore, it is worth noting that there is a prevalent occurrence of elongated axial spheroids and flattened corneas. Other notable characteristics that can be observed in the skin include the skin striatum, and lung lesions such as the pulmonary air bag that causes spontaneous pneumothorax. The tissues affected by MFS demonstrate characteristics that align with the distribution and prevalence of FBN1 (Schrenk et al., 2018; Du et al., 2021).

Notably, MFS has a prominent impact on the cardiovascular system, which manifests as mitral valve prolapse and regurgitation, as well as aortic aneurysm and aortic coarctation. Aortic aneurysm and aortic constriction are serious and potentially fatal consequences associated with MFS (Schrenk et al., 2018; Du et al., 2021). FBN1 plays a critical role in serving as organizing scaffolds during the process of elastic fiber production and maturation (Milleron et al., 2020; de la Fuente-Alonso et al., 2021). In the MFS (Fbn1^C1039G/+^) mouse model, atypical elastic fibers alter the carrying amplitude of the aorta, leading to micro-dissection and denaturation of the media in the aorta (Habashi et al., 2006). Aortic aneurysm is distinguished by impaired functionality of vascular cells, compromised integrity of ECM, and diminished mechanical properties of the vascular wall. Studies have demonstrated that the advancement of thoracic aortic aneurysm (TAA) in MFS is associated with the upregulation of MMP-2 and MMP-9, which are implicated in the substantial degradation of elastic fibers, alterations in aortic mechanical characteristics, impairment of endothelial function, and diminishment of smooth muscle contractility (Chung et al., 2007a; Chung et al., 2007b; Chung et al., 2008a; Chung et al., 2008b; Asano et al., 2022; Lim et al., 2022). Antagonism of TGF-β by neutralizing antibody or losartan, an AT1R blocker, proved to normalize the expression and stimulation of MMP-2 and MMP-9 in MFS (Fbn1^C1039G/+^) mouse model and to prevent the formation of TAA. In addition, losartan has been shown to ameliorate microfiber deposition in fibroblasts from Geleophysic dysplasia type 2 (Piccolo et al., 2019), which is characterized by an opposite clinical phenotype compared to MFS. Other studies using Fbn1^C1039G/+^ mice show that TGF-β signaling via ERK1/2 pathway is the main driving factor for the formation of TAA, which could be inhibited by AT2R-mediated angiotensin II signaling (Holm et al., 2011). Nevertheless, the function of TGF-β in the development of TAA remains a subject of debate and uncertainty. Losartan has also been utilized in human subjects as a therapeutic intervention for the management of TAA in MFS through several clinical trials(Brooke et al., 2008; Möberg et al., 2012; Milleron et al., 2015; Sandor et al., 2015; Forteza et al., 2016). A study employing a randomized, double-blind, placebo-controlled design demonstrated that losartan effectively lowered blood pressure in individuals with MFS, although it did not exhibit a reduction in aortic dilatation, contrary to findings observed in mouse models (Milleron et al., 2015). Furthermore, when compared to atenolol, losartan did not yield significant alterations in aortic root and ascending aortic diameters during a 3-year follow-up period (Forteza et al., 2016). Two studies independently report that the genetic inhibition of TGF-β signaling in postnatal smooth muscle cells in Fbn1^C1039G/+^ mice aggravates, rather than alleviates, TAA (Li et al., 2014; Wei et al., 2017), implying that TGF-β could have a protective function in the evolution of TAA, but its mechanism remains to be clarified. These results suggest the complexity of the TGF-β signaling in the pathogenesis of MFS. However, available evidence suggests that the inhibition of TGF-β has not yielded satisfactory therapeutic outcomes for individuals with MFS.

In the myocardial pathology, the primary presentation of cardiac lesions in MFS mice is dilated cardiomyopathy (DCM). The occurrence of DCM is contingent upon the insufficiency of FBN1 inside the pericellular matrix of the myocardium (Cook et al., 2014). The absence of FBN1 results in a decrease in the mechanical robustness and structural stability of the myocardium, leading to impaired muscle contractility. Cardiomyocytes lacking FBN1 demonstrate aberrant modulation of cardiac mechanosensors, specifically the AT1 receptor (AT1R) and β1 integrin, leading to an elevated β-arrestin-2-promoted ERK1/2 and weakened focal adhesion kinase (FAK) signaling. Furthermore, crosstalk may take place between AT1R and β1 integrin in FBN1-deficient cardiomyocytes (Cook et al., 2014; Campens et al., 2015). AT1R antagonist restores the size and function of MFS heart, underscoring the pivotal function of FBN1 across the myocardial pathology of MFS (Cook et al., 2014).

There exists an association between MFS and related disorders with over 400 distinct variants identified in FBN1 (Collod-Béroud et al., 2003). At present, there are more than 3000 FBN1 mutations known, more than 1800 genetic anomalies have been found in the whole length of FBN1 (Collod-Béroud et al., 2003). Of these, point mutations are the prevailing form of mutation, constituting 66.3% of all documented mutations. Deletions rank second, comprising 16.1% of mutations, while splice site mutations account for 10.9%. Insertions and duplications are less frequently observed, accounting for 5.4% and 0.2%, respectively. Point mutations can be categorized into two main types: nonsense mutations, which account for approximately 17.1% of all point mutations, and missense mutations, which make up the remaining 82.9% of point mutations (Zeyer and Reinhardt, 2015). The most prevalent form of FBN1 mutations is represented by missense mutations, the majority of which are cysteine substitutions (Coelho and Almeida, 2020; Du et al., 2021). Approximately 73.1% of all missense mutations occur in the cbEGF structural domain, which is usually disrupted by substitution or insertion of a cysteine that is essential for normal folding, or by altering a residue that binds calcium, or by changing a glycine that has no defined function (Zeyer and Reinhardt, 2015; Milewicz et al., 2021).

There have been two proposed working models about the underlying causes of MFS. FBN1 alterations can be categorized into 2 distinct groups, namely, dominant negative (DN) and haploinsufficiency (HI). DN FBN1 mutations result in the synthesis and secretion of structurally compromised FBN1 that interferes with the functionally normal FBN1 from the non-mutant allele. This affects the function of protein folding as well as protein-protein interactions, leading to abnormal ECM network (Franken et al., 2016; Mannucci et al., 2020). However, it is crucial to acknowledge that there are several pathways such as nonsense-mediated mRNA degradation or under-secretion, resulting in reduction of FBN1 expression. When the amount of normal FBN1 in the ECM is lowered, it results in the inability of the ECM to function properly (Mannucci et al., 2020; Muthu and Reinhardt, 2020). Notably, mutations resulting in reduced FBN1 expression correlate directly with disease severity (Park et al., 2017). Therefore, it is more likely that reduced levels of FBN1 lead to reduced levels of normal microfibers in the tissues, weakening the integrity of the ECM, causing structural disorders that mediate the phenotype of MFS (Park et al., 2017; Muthu and Reinhardt, 2020).

Due to the importance of FBN1 microfibrils in modulating the bioavailability of TGF-β, this could be alternative mechanisms leading to the pathogenesis of MFS (Baudhuin et al., 2015; Takeda et al., 2018; Verhagen et al., 2021). An increase in TGF-β signaling was confirmed in mouse models with FBN1 mutations. Of particular importance, the amelioration of abnormalities related to FBN1 deficient mice can be achieved with the administration of TGF-β antagonists, including TGF-β neutralizing antibodies and angiotensin II type 1 (AT1) receptor blockers (Ng et al., 2004; Habashi et al., 2006; Le Goff and Cormier-Daire, 2012). Interestingly, a study reported that the acceleration of abnormal aortic growth and rupture was associated with deficiencies in the expression of angiotensin II type 2 (AT2) receptors. The study demonstrated that the selective blocker of AT1 receptors, losartan, inhibited the TGF-β-mediated activation of ERK by allowing continued signaling through AT2 (Habashi et al., 2011). FBN1 interacts and sequesters TGF-β, whereby it modulates the bioavailability of TGF-β. Therefore, FBN1 decrease or deficiency results in a failure of TGF-β sequestration, leading to an increase of TGF-β levels. Upon activation of TGF-β signaling, the creation of a complex between Smad2/3 and Smad4 occurs, resulting in the translocation of this complex to the nucleus and subsequent stimulation of transcription for certain target genes. At the same time, induction of TGF-β signaling pathway results in an upregulation of MMPs, enzymes that are responsible for the degradation of ECM proteins such as FBN1 and the activation and release of ECM bound growth factors (Furlan et al., 2021; Zimmermann et al., 2021). This process contributes to the worsening of ECM instability and aggravating disease phenotype (Liu et al., 2014; Takeda et al., 2018; Cale et al., 2021).

## 7 *FBN1* mutations and other genetic diseases

Mounting evidence shows that not all mutations occurring in the *FBN1* gene lead to MFS. Alterations in *FBN1* gene also lead to MASS (mitral valve, myopia, aorta, skin and skeletal condition) phenotype (Fusco et al., 2019), Marfanoid-progeroid-lipodystrophy, Acromic dysplasia, Stiff skin syndrome (SSS), Geleoplastic dysplasia, Weill-Marchesani syndrome (WMS) (Sengle et al., 2012; Sakai and Keene, 2019; Al Motawa et al., 2021; Marzin et al., 2021), which are not related to MFS (Loeys et al., 2010a). Table 2 lists the genetic diseases caused by FBN1 mutations in patients.

**TABLE 2:** Human genetic diseases due to mutations in gene encoding FBN1.

Syndrome	Disease gene	Phenotype	OMIM number
Marfan syndrome	*FBN1*	Elevated height, disproportionately long limbs and digits, mitral valve prolapse, mitral regurgitation, dilatation of the aortic root, myopia, aortic regurgitation, elevated axial globe length, corneal flatness	154,700
Ectopia lentis 1	*FBN1*	Ectopia lentis	129,600
Marfanoid-progeroid-lipodystrophy syndrome	*FBN1*	Congenital lipodystrophy, premature birth with an accelerated linear growth disproportionate to weight gain, progeroid appearance with different facial properties	616,914
Stiff skin syndrome	*FBN1*	Thickened and indurated skin of the whole body, restriction of joint mobility with flexion contractures	184,900
Acromicric dysplasia	*FBN1*	Serious short stature, short hands and feet, joint restriction, skin thickening, round face, well-defined eyebrows, hoarse voice and pseudomuscular build	102,370
Weill-Marchesani syndrome 2	*FBN1*	Short stature, brachydactyly, joint stiffness, and lens alterations	608,328
MASS syndrome	*FBN1*	Long limbs, striae atrophicae, deformity of the thoracic cage, mitral valve prolapse, mild dilatation of the aortic root	604,308
Geleophysic dysplasia 2	*FBN1*	Serious short stature, short hands and feet, joint restriction, and skin thickening, a ‘happy’ face with full cheeks, progressive cardiac valvular thickening	614,185

Specifically, SSS is a pathological dermatofibrosarcoma that arises due to mutations occurring in the TB4 structural domain of FBN1. Clinically, this condition is distinguished by the excessive accumulation of microfibers and fibrosis in the skin. However, it does not exhibit the characteristic manifestations of bone overgrowth, lens dislocation, and aortic aneurysm that are commonly observed in individuals with MFS (Loeys et al., 2010b). As previously stated, TB4 encodes the RGD motif, which exhibits binding affinity towards various integrins. It has been reported that at the cellular level, the TB4 structural domain of mutant FBN1, resembling SSS, can influence integrin-mediated cellular adhesion, which manifests a substantial reduction in adhesion mediated by integrins α5β1, αvβ5, and αvβ6, as well as a partial decrease in adhesion mediated by αvβ1 (Del Cid et al., 2019). Furthermore, activation of β1 integrin or blockade of β3 integrin in mouse models of SSS has an important role in symptomatic improvement of SSS (Gerber et al., 2013). This indicates that the abnormal binding of integrins, resulting from mutations in the TB4 structural domain, plays a crucial role in the development of SSS. Furthermore, the enhanced signaling of TGF-β also contributes to the progression of SSS (Loeys et al., 2010b). It was found that enhanced TGF-β signaling in SSS may be related to integrins, as some integrins can directly mediate TGF-β activation (Loeys et al., 2010b; Munger and Sheppard, 2011). Consequently, the anomalous interaction between mutated FBN1 and integrins, along with the formation of atypical microfibers in SSS, may synergistically initiate aberrant TGF-β activation, thereby contributing to the progression of the disease.

Mutations in the *FBN1* gene not only cause MFS with tall stature and joint laxity, but also cause the opposite manifestation, which is mainly seen in Weill-Marchesani syndrome (WMS), Geleophysic dysplasia (GD) and Acromelic dysplasias (AD). The common features of these three disorders are severe short stature, short limbs and stiff joints (Cecchi et al., 2013; Cheng et al., 2018). The missense mutations in this group of diseases are located downstream of TB4, the heparin-binding TB structural domain (TB5), and mutations at this position affect binding to heparin (Le Goff et al., 2011; Cain et al., 2012). The abnormal binding of FBN1 with heparin may have led to the assembly and deposition of microfibril, further contributing to the associated clinical symptoms. However, the question remains how mutations in *FBN1* at this location lead to clinical features opposite to MFS, and more studies are needed to clarify the specific pathological mechanisms.

## 8 Role of FBN1 in tumorigenesis

Dysregulation of FBN1 has been shown to be associated with tumorigenesis (Table 1). In gastric cancer, succinylation modification of FBN1 leads to its accumulation, activates TGF-β1 and triggers the activation of the phosphoinositide 3-kinase (PI3K)/AKT signaling, thereby promoting tumor proliferation (Wang X. et al., 2022). FBN1 is also regulated by microRNA in gastric cancer. Because FBN1 is a direct target of miR-133b, downregulation of miR-133b in gastric cancer leads to the upregulation of FBN1 expression, which promotes the proliferation, migration and invasive ability of gastric cancer cells (Yang et al., 2017). FBN1 is closely related to colorectal cancer, and studies suggest that FBN1 methylation is an important biomarker for monitoring colorectal cancer progression. The detection of hypermethylated FBN1 in stool samples is a non-invasive and useful method for screening colorectal cancer (Guo et al., 2013).

A high level of FBN1 may also promote osteosarcoma invasion, migration, and progression (Liu et al., 2020). Silencing lncRNA-PGM5-AS1 increases miR-140-5p expression, which in turn reduces FBN1 levels, thereby attenuating *in vitro* osteosarcoma epithelial-mesenchymal transition (EMT), invasion and migration, and *in vivo* tumorigenesis (Liu et al., 2020). FBN1 is also closely associated with papillary thyroid carcinoma. It is highly expressed in papillary thyroid carcinoma, and silencing of FBN1 inhibits cell viability and colony formation *in vitro* and inhibits tumor growth *in vivo* (Ma et al., 2016). In addition, FBN1 is also a key gene associated with the prognosis of squamous cell carcinoma through a comprehensive analysis of multi-omics data (Feng et al., 2021). In renal clear cell carcinoma, microarray analysis reveals that FBN1 induction is accompanied by a higher malignancy grade and progression. Furthermore, high expression of FBN1 is associated with poor survival outcome in renal clear cell carcinoma (Chen et al., 2017).

In ovarian cancer, RNA-seq demonstrates that FBN1 is highly expressed in cisplatin-resistant ovarian cancer. Studies show that FBN1 regulates glycolysis and angiogenesis through activation of the vascular endothelial growth factor receptor 2 (VEGFR-2)/STAT2 pathway, decreases the sensitivity of ovarian cancer to cisplatin and promotes chemoresistance in ovarian cancer (Wang Z. et al., 2022). Meanwhile, FBN1 has been identified as a prospective biomarker to assess overall survival and progression-free survival in ovarian cancer patients (Chen et al., 2020). Consistently, there is a significant correlation between FBN1 and ovarian cancer prognosis, and high level of FBN1 leads to poor prognosis in ovarian cancer patients (Zuo et al., 2021). However, in endometrial carcinoma, the expression of FBN1 is downregulated. Taken together, as shown in Table 1, the expression of FBN1 in tumors and its mechanism of action may depend on the origin and type of tumors, and the specific mechanism deserves to be further investigated.

## 9 FBN1 and kidney diseases

We recently found that FBN1 exhibits significant upregulation in the decellularized kidney tissue scaffold (KTS) in CKD (Li et al., 2021; Li et al., 2022; Li et al., 2023; Peng et al., 2023). Furthermore, FBN1 expression is elevated across many animal models of CKD as well as in individuals diagnosed with CKD. Of interest, FBN1 is primarily induced in the tubular epithelial cells in CKD, but not in interstitial fibroblasts. In addition, serum FBN1 level is elevated and closely correlated with the severity and stage of CKD. There exists an adverse association between the amount of serum FBN1 and the estimated glomerular filtration rate (eGFR), while a positive correlation is shown between serum FBN1 and serum creatinine, blood urea nitrogen (BUN), and cystatin C (Li et al., 2021).

In order to investigate the impact of FBN1 on kidney, the renal phenotype of mice with under-expressed FBN1 is examined within the context of the MFS model. Mesangial area and glomerular volume are significantly decreased in glomeruli of FBN1-underexpressing mice (Hartner et al., 2004; de Souza et al., 2023). FBN1 exists in the mesangial matrix of the glomerulus, and its expression is induced in the acute phase of anti-Thy1.1 glomerulonephritis (Porst et al., 2006). FBN1 plays an essential role in the maintenance of mechanical stability and elasticity in glomerular capillaries. FBN1 facilitates the processes of adhesion, migration, and progression of mesangial cells, which may lead to excessive mesangial expansion during glomerular diseases (Porst et al., 2006). In addition, within the glomerulus basement membrane regions, FBN1 can bind to fibulin-2 to stabilize the interaction between microfibrils and the lamina densa (Reinhardt et al., 1996). FBN1 may cause glomerular damage in hypertension and diabetic nephropathy (Porst et al., 2006). Mice with hypertension induced by DOCA salt or diabetes induced by streptozotocin (STZ) exhibit a significant increase in glomerular FBN1 deposition (Hartner et al., 2006).

**FIGURE 4:**
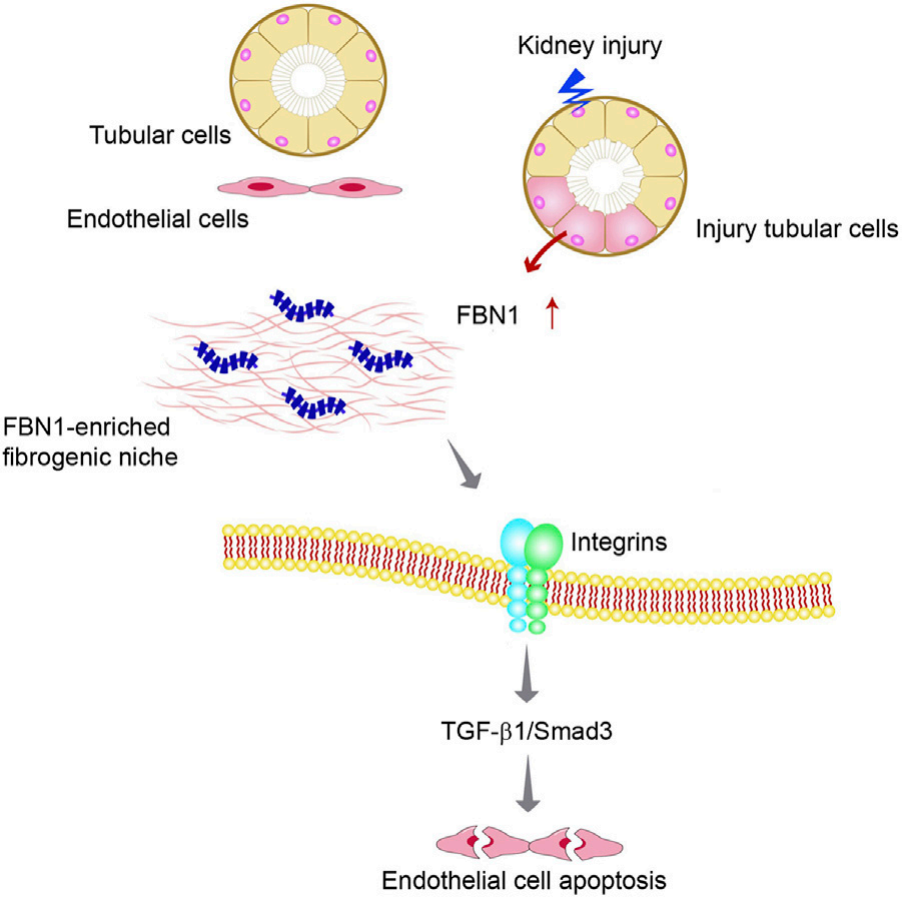
The fate of peritubular endothelial cells is controlled by the tubule-derived FBN1 in chronic kidney disease (CKD). Diagram shows that injured tubular cells produce and secrete FBN1, which orchestrates the formation of a hostile FBN1-enriched microenvironment for endothelial cells. FBN1 then triggers endothelial cell apoptosis by a cascade of integrins/TGF-β1/Smad3 signaling.

Recent studies suggest that FBN1 is an integral component of the fibrogenic niche formed in CKD (Li et al., 2021). Furthermore, it is interesting to note that the FBN1-enriched niche renders an unfavorable condition for endothelial cells and triggers apoptosis in these cells through the integrin αvβ6/TGF-β1/Smad3 signaling pathway (Figure 4). *In vitro*, FBN1 induces apoptosis of endothelial cells and inhibits their proliferation. Within the context of an experimental mouse model of CKD, depletion of FBN1 has been observed to improve renal fibrotic lesions and alleviate vascular rarefaction (Li et al., 2021). These studies provide evidence that FBN1 plays a crucial role in facilitating vascular rarefaction by orchestrating an unfavorable microenvironment for endothelial cells.

## 10 Role of FBN1 in other organ systems

FBN1 has been identified as an important regulator of adipose tissue development. Studies show that FBN1 level in mice is related to the size of adipose tissue. In mice fed with a high-fat diet, the mRNA level of FBN1 is increased in white adipose tissue (Muthu and Reinhardt, 2020). In humans, the mRNA of FBN1 in adipose tissue of obese women is upregulated, which is related to the increase of adipocyte size (Davis et al., 2016), suggesting a correlation between FBN1 levels and adiposity. In addition, FBN1 can regulate adipose tissue development and function (Muthu et al., 2022). It can negatively regulate adipose differentiation and maintain adipose tissue homeostasis by inhibiting the insulin signaling (Muthu et al., 2022). FBN1 is also involved in the transformation of undifferentiated mesenchymal stem cells into adipose, but its expression is downregulated with the development and maturation of adipocytes (Muthu et al., 2022). However, FBN1 has little contribution to lipid storage and metabolic homeostasis. The mutations or loss of FBN1 are not sufficient to disrupt TGF-β signaling in adipose tissue or to mimic the clinically observed adipose and muscle dysplasia (Walji et al., 2016).

In the skeletal system, fibrillins are expressed in long bones, ribs, vertebral bodies and cartilage, and FBN1 is expressed and secreted by differentiating osteoblasts (Tiedemann et al., 2013). FBN1 regulates osteogenic/adipogenic lineage selection of bone marrow mesenchymal stem cells through IL4Rα/mTOR signaling. The inhibition of mTOR cascade by rapamycin ameliorates the osteopenia phenotype in *Fbn1* ^*+/−*^ systemic sclerosis (SSc) mice (Chen et al., 2015). FBN1 can also regulate mesenchymal stem cell activity by regulating the bioavailability of TGF-β and BMP in the microenvironment of the bone marrow niche (Smaldone et al., 2016b; Delhon et al., 2022). Furthermore, FBN1 is upregulated in patients with fatal osteogenesis imperfecta, causing abnormal accumulation of TGF-β and BMP in the ECM, resulting in defects of osteoblasts and matrix (Bini et al., 2021).

In the hematologic system, FBN1 differentially regulates TGF-β signaling in the hematopoietic stem cell and erythroid niches. FBN1 promotes the expansion of hematopoietic stem cells but limits the expansion of erythrocytes. The deletion of FBN1 in mouse bone marrow leads to significant hematopoietic abnormalities, resulting in depletion of hematopoietic stem cells and increased erythropoiesis (Smaldone et al., 2016a).

FBN1 is also associated with idiopathic pulmonary fibrosis. Proteomic analysis reveals that FBN1 is a key protein in extracellular capsule cargo associated with idiopathic pulmonary fibrosis and may play a vital role in the progression of idiopathic pulmonary fibrosis (Velázquez-Enríquez et al., 2021). FBN1 has also been found to be closely related to injury repair. It may promote myofibroblast transdifferentiation by regulating TGF-β signaling and participates in wound healing process by regulating cell adhesion through integrins (Zhang et al., 2022). In human dental pulp wound healing, downregulation of FBN1 and enhanced protein degradation affect wound healing and the formation of mineralized tissue barrier (Yoshiba et al., 2012).

## 11 FBN1 and other signaling pathway

Studies have revealed that FBN1 has a regulatory effect on ERK1/2 signaling pathway. Most genetic diseases such as MFS, SSS and SSc caused by dysregulated FBN1 exhibit an upregulated ERK1/2 signaling (Holm et al., 2011; Gerber et al., 2013; Lim et al., 2022; Rokni et al., 2022). FBN1 can activate ERK1/2 signal pathway through RAS-RAF-MEK1/2-ERK1/2 signal cascade. Furthermore, the RGD motif of FBN1 inhibits the expression of miR-1208 via c-Src kinase and its downstream JNK signaling. The suppression of miR-1208 leads to elevated levels of both total and phosphorylated ERK1/2 and MEK1/2 proteins, together with an increase in the ratio of phosphorylated to total ERK1/2 (Zhang et al., 2021).

FBN1 also phosphorylates the VEGFR2 at Tyr1054 residue, leading to the activation of its downstream focal adhesion kinase (FAK)/protein kinase B (PKB or AKT) pathway. This in turn leads to phosphorylation of the tyrosine residue 690 (Tyr691) on STAT2, promotes its nuclear translocation, and finally alters the expression of genes related to angiogenesis and glycolysis mediated by STAT2 (Wang Z. et al., 2022). In addition, FBN1 may also mediate the metastasis of ovarian cancer by the p53 signaling (Wang et al., 2015). Collectively, FBN1 may modulate a variety of biological processes through multiple signal pathways.

## 12 Conclusion

FBN1, an ECM glycoprotein that serves as a structural component of calcium binding microfibrils, plays a fundamental role in providing force bearing structural support in elastic and nonelastic connective tissue throughout the body. As such, mutations in *FBN1* gene cause a wide variety of genetic disorders with pleiotropic manifestations (Table 2). As an ECM protein, FBN1 also interacts with many microfibril-associated proteins, growth factors and cell membrane receptors, thereby regulating a diverse array of biological processes such as cell growth, migration, apoptosis and differentiation. In this context, dysregulation of FBN1 expression has been associated with the pathogenesis of various human diseases including cancer, cardiovascular and kidney disorders.

While much has been learned about the connection and mechanistic interplay between *FBN1* gene mutation and various genetic diseases, the role of dysregulated FBN1 in the pathogenesis of different diseases is just beginning to be unveiled. Given that both reduction of FBN1 due to gene mutations and upregulation of FBN1 in various diseased conditions paradoxically activate TGF-β signaling (Figure 3), it is conceivable that the action of FBN1 may be context-dependent in different settings. Therefore, future studies are warranted to delineate the specific interaction of FBN1 with other factors and unravel new mechanisms of FBN1 in different settings. A better elucidation of FBN1 specific action will help to understand the logic behind various human diseases and identify novel therapeutic target for intervention.
